# Chitosan oligosaccharide affects antioxidant defense capacity and placental amino acids transport of sows

**DOI:** 10.1186/s12917-016-0872-8

**Published:** 2016-11-02

**Authors:** Chunyan Xie, Xin Wu, Cimin Long, Qinhua Wang, Zhiyong Fan, Siming Li, Yulong Yin

**Affiliations:** 1 Key Laboratory of Agro-ecological Processes in Subtropical Region, Institute of Subtropical Agriculture, Chinese Academy of Sciences, Changsha, Hunan 410125 China; 2 Hunan Provincial Engineering Research Center of Healthy Livestock and Poultry Production, Changsha, 410125 Hunan China; 3 Hunan Co-Innovation Center of Animal Production Safety, CICAPS, Hunan Agricultural University, Changsha, 410128 China; 4 Institute of Animal Science, Jiangxi Academy of Agricultural Sciences, Nanchang, 330200 China

**Keywords:** Chitosan oligosaccharides, Antioxidant defense, Amino acids transport, Sow

## Abstract

**Background:**

Chitosan oligosaccharide (COS) is widely consumed as a functional food due to its multiple health effects, but few studies about COS supplement on placental antioxidant and nutrition transport capacity were reported. Taken pregnant sow as a model, we aimed to investigate the effects of dietary COS supplementation during late gestation on placental amino acids transport and antioxidant defense capacity of sows. From day (d) 85 of gestation to parturition, sixteen pregnant sows were divided into a control group (basal diet without COS supplementation) and a COS group (30 mg COS/kg basal diet). Plasma sample of sow was collected on d 110 of gestation, and placenta tissue was obtained during parturition. Then plasma antioxidant enzyme’s activities, the relative level of oxidant stress related genes, amino acids transport related genes and mTOR pathway molecules in placenta were determined.

**Results:**

Results showed that maternal dietary supplementation with COS increased (*P* < 0.05) plasma total SOD, caused a downtrend in plasma MDA (0.05 < *P* < 0.10) on d 110 of gestation. Interestingly, the mRNA expression of some antioxidant genes in the placenta were increased (*P* < 0.05) and pro-inflammatory cytokines were reduced (*P* < 0.05) by COS supplement, whereas no significant difference was observed in the activities of placental total SOD and CAT between two groups. Additionally, further study demonstrated that COS feeding stimulated mTOR signaling pathway, increased amino acids transporters expression in placenta.

**Conclusions:**

These observations suggested that COS supplement in sow’s diet during late gestation enhanced antioxidant defense capacity of sows, promoted placental amino acids transport, which may contribute to the health of sows and development of fetus during gestation.

## Background

Pregnancy is a state of oxidative stress arising from increased placental mitochondrial activity and production of reactive oxygen species (ROS), including nitric oxide, carbon monoxide, and peroxynitrite, which have pronounced effects on placental function including trophoblast proliferation and differentiation and vascular reactivity [1]. The oxidant/antioxidant status affects the entire unit mother-placenta-fetus, but the disturbance of which during pregnancy may be one of the key downstream mediators that initiate programming of the offspring [2]. Excessive free radical production may cause both lipid and protein oxidation and impair normal endothelial cell function [3]. In this regard, oxidative stress and/or low intake of antioxidant involved in the etiopathogenesis of the most frequent disorders in the gestational period, which was closely related to reproductive performance of sows [4]. Consequently, supplementation with antioxidant in sow’s diet during late gestation and lactation periods is necessary.

As a natural polysaccharide, COS is the depolymerized product of chitosan, and has good solubility in water and various biological activities, including immune-stimulant [5], anti-inflammatory [6] and antioxidant properties [7]. Numerous studies have shown that chitosan has beneficial effects on relieving the oxidative stress [8]. Moreover, placenta, as the main interface between the mother and the fetus, has many functions to perform, including producing and secrete hormones and cytokines, and mediatting the transfer of nutrients, oxygen and waste products [9]. Nutrients pass from the maternal to the fetal blood across the syncytiotrophoblast and the endothelium of the fetal capillaries, therefore main determinant of fetal growth is placental nutrient transport, which is essential for fetal growth and development [10]. Jansson et al. (2006) found the pregnant rats subjected to protein malnutrition during gestation down-regulated placental amino acid transport, which may contribute directly to the development of IUGR [11].

Our previous study has found that maternal COS supplement increased birth weight and growth rate of piglets [12], and postpartum upregulates cholesterol accumulation in suckling piglets [13]. Therefore, we hypothesised that supplementation with COS in sow diets could affect the function of the placenta. The aim of the present study was to investigate the effects of maternal supplementation with COS during late gestation on the antioxidant defense capacity and the amino acids transport in placenta.

## Methods

### Animals and experimental treatment

Sixteen pregnant sows (Large White × Landrace) with the same parturition history were obtained from an experimental livestock farm (Twins, Inc., JiangXi, China), and fed one of two experimental diets from 85 days of gestation to parturition (8 sows/diet). The experiment last for about 30 days. Sows were randomly divided into two groups, 1) all sows were fed with basal diet (Control) (*n* =20), 2) all sows were fed with basal diet containing 0.03 ‰ (wt/wt) COS (ZTH Tech. Co., Beijing, China), and the dose of chitosan oligosaccharides was determined by preliminary experiment [14]. The gestation diet formulations have been formulated to meet or exceed all the requirements for gestating sows as referred in our previous study [15]. Sows had free access to water at all times. The diet composition was list in Table 1.

**Table 1:** Composition of gestation and lactation diet (as-fed basis)

Item	Control	COS	Item	Control	COS
Ingredient (%)			Nutrient composition		
Corn	70.30	70.30	DM, %	88.2	88.2
Soybean meal	12.00	12.00	ME, MJ/kg	13.5	13.5
Wheat middling	4.00	4.00	CP, %	14.7	14.7
Rice bran meal	3.00	3.00	Lys, %	0.78	0.78
Puffed soybean	3.00	3.00	Met + Cys, %	0.69	0.69
Salt	0.35	0.35	Ca, %	0.92	0.92
Potassium chloride	0.75	0.75	Total P, %	0.67	0.67
Vitamin-mineral Premix^a^	3.00	3.00			
Dicalcium phosphate	2.20	2.20			
Limestone	0.50	0.50			
Soy oil	0.50	0.50			
Mold-inhibiter	0.09	0.09			
DL-Met	0.06	0.06			
L-Thr	0.05	0.05			

^a^Premix provided the following per kg of diet: Fe (FeSO_4_ · H_2_O), 80 mg; Mn (MnSO_4_ · 5H_2_O), 45 mg; Zn (ZnO), 100 mg; Cu (CuSO_4_ · 5H_2_O), 20 mg; I (KI), 0.70 mg; Se (Na_2_SeO_3_ · H_2_O), 0.25 mg; vitamin A, 10,000 IU; vitamin D_3_, 2,500 IU; vitamin E, 100 IU; vitamin K, 10 IU; vitamin B_2_, 10 mg; vitamin B_6_, 1 mg; vitamin B_12_ 50ug; biotin, 80ug; folic acid, 5 mg; nicotinic acid, 15 mg; choline chloride 1500 mg

The degree of polymerization of COS used in this experiment was 2–7 and was purchased from Zhong Tai He technology co., LTD.

### Sample collection

In this study, eight sows/group were randomly selected for sample collection. On d 110 of gestation, a five-mL blood sample of sow was collected from the ear vein. Plasma sample was then obtained by centrifugation at 3,000 × g for 10 min at 4 °C and immediately stored at –80 °C for antioxidant analysis [16]. During parturation, a part of placenta/sow tissue was obtained and immediately frozen in liquid nitrogen and stored at –80 °C until required for analysis.

### Measurement of antioxidant enzyme’s activities or oxidant injury product

Plasma glutathione peroxidase (GSH-Px), total superoxide dismutase (T-SOD), catalase (CAT) and malondialdehyde (MDA) in the placenta and plasma were determined by assay kits (Nanjing Jiancheng Biotechnology Institute, Nanjing, China) according to the manufacturer’s instructions.

About 0.5 g placenta tissue was powered in liquid nitrogen and dissolved in 5 mL PBS solution. The total superoxide dismutase and catalase in the supernatant of placenta were determined by ELISA kits (BlueGene, Shanghai, China) according to the manufacturer’s instructions. All samples were measured by UV/visible spectrophotometer (UV-2450, Shimadzu, Kyoto, Japan).

### RNA isolation and real-time polymerase chain reaction

Total RNA of placenta was isolated using TRIzol reagent (Life Technologies, Tokyo, Japan) according to the manufacturer’s protocol. The concentration of total RNA in each sample was quantified spectrophotometrically at 260 nm and established by 260/280 values to assess RNA quality. The mRNA expression of placental genes and β-actin were quantified by SYBR real-time polymerase chain reaction (PCR) (SYBR Premix Ex Taq; Takara bio Inc., Shiga, Japan). Primers were designed with Primer 5.0 according to the gene sequence of pigs (http://www.ncbi.nlm.nih.gov/pubmed/) to produce an amplification product (Table 2). The amplification reactions were carried out in an ABI Prism 7900 HT sequence detection system (Applied Biosystems, Foster, CA). The relative level of mRNA expression was calculated using the 2^−ΔΔCt^ method after normalization with β-actin as a housekeeping gene.

**Table 2:** Primers used for real-time PCR analyzing

Genes	Nucleotide sequence of primers (5’–3’)	Size (bp)	Ref.
Gpx1	F: TGGGGAGATCCTGAATTG R: GATAAACTTGGGGTCGGT	183	[19]
Gpx4	F: GATTCTGGCCTTCCCTTGC R: TCCCCTTGGGCTGGACTTT	172	
Ucp2	F: CCAATGTCGCTCGTAATG R: TGGCAGGGAAGGTCATC	109	
p53	F: CTGCTTCCTGAAAACAACC R: AAGGGACAAAGGACGACA	199	
Mn-SOD	F: GGACAAATCTGAGCCCTAACG R: CCTTGTTGAAACCGAGCC	159	
Cu/Zn-SOD	F: CAGGTCCTCACTTCAATCC R: CCAAACGACTTCCASCAT	255	
CAT	F: CGAAGGCGAAGGTGTTTG R: AGTGTGCGATCCATATCC	374	This study
Hsp70	F: GCCCTGAATCCGCAGAATA R: TCCCCACGGTAGGAAACG	152	[17]
TNFα	F: CCACGTTGTAGCCAATGTCA R: CAGCAAAGTCCAGATAGTCG	395	
IL-6	F: TCAGTCCAGTCGCCTTCTCC R: GGCATTTGTGGTGGGGTTAG	494	
IL-8	F: CTGGCTGTTGCCTTCTTG R: TCGTGGAATGCGTATTTATG	113	
IL-1β	F: GCTAACTACGGTGACAACAA R: TCTTCATCGGCTTCTCCACT	196	
EAAT1	F: GATGGGACCGCCCTCTAT R: CGTGGCTGTGATGCTGATG	105	This study
EAAT2	F: GGCTGCTGGACAGGATGA R: TAAATGGACTGGGTCTTGGT	154	This study
PAT1	F: TGTGGACTTCTTCCTGATTGTC R: CATTGTTGTGGCAGTTATTGGT	125	This study
PAT2	F: GGGCTACTTGCGGTTCGG R: GCGCTTTGACACCTGGGAG	181	This study
LAT1	F: TTTGTTATGCGGAACTGG R: AAAGGTGATGGCAATGAC	155	This study
ASCT2	F: GATTGTGGAGATGGAGGATGTGG R: GCGAGTGAAGAGGAAGTAGATGA	128	
PEPT1	F: CATCGCCATACCCTTCTG R: TTCCCATCCATCGTGACATT	143	
SNAT2	F: TACTTGGTTCTGCTGGTGTCC R: GTTGTGGGCTGTGTAAAGGTG	212	This study
VEGF-A	F: CAACGACGAAGGTCTGGAGTG R: GCCTCGCTCTATCTTTCTTTGG	155	[15]
β-actin	F: CGTTGGCTGGTTGAGAATC R: CGGCAAGACAGAAATGACAA	132	This study

### Total protein extraction and western blotting

Placental tissue were pulverized in liquid nitrogen and lysed in RIPA buffer (Beyotime Biotechnology, China) plus 1 mM PMSF and 1 % phosphatase inhibitor. After the sample was centrifuged at 12,000 × g, 4 °C, for 10 min, then the protein concentration in the supernatant was determined using a Bicinchoninic Acid assay (Beyotime Biotechnology, China).

Western blotting were performed as described previously [17]. The membranes were incubated with primary antibodies, including total mTOR (#2972; Cell Signaling Technology), p-mTOR (#5536; Cell Signaling Technology), p-4EBP1 (#9451; Cell Signaling Technology), p-p70S6K (ab126818; Abcam) and β-actin (sc-47778; Santa Cruz) antibodys at 1:1000 dilution. After washed with TBST, the membranes were incubated for 2 h with horseradish peroxidase-linked secondary antibodies (Beijing ZhongShan Golden Bridge Biological Technology Co., LTD, China). Finally, the membranes were washed with TBST, and then developed using Supersignal West Dura Extended Duration Substrate according to the manufacturer’s instructions (Pierce, Rockford, IL). Western blots images were quantified by measuring the intensity of correctly sized bands using Alpha Imager 2200 (Alpha Innotech Corporation, CA, USA) software, and all protein measurements were normalized to β-actin.

### Statistical analysis

Statistical analyses were carried out using a t test, SPSS statistics 17. All the results are expressed as means with their standard errors (SPSS Institute, Inc.). Differences were considered to be statistically significant, and probability *P* values < 0.05 were taken to indicate statistical significance, and probability values between 0.05 and 0.10 were considered to be trends.

## Results

### Plasma antioxidant enzymes’ activities and oxidant injury product

To investigate the effects of supplementation with COS during late gestation on anti-oxidant capacity, plasma GSH-Px, total-SOD and CAT were analyzed (Table 3). The total SOD activity in the COS group was increased (*P* < 0.05), whereas plasma GSH-Px and CAT weren’t affected on d 110 of gestation compared with that in the control group (*P* > 0.10). The most common product of lipid peroxidation MDA, as an oxidative damage level of sows, also presented the downtrend in the COS group on d 110 of gestation compared with that in the control group (0.05 < *P* < 0.10).

**Table 3:** Plasma antioxidant enzyme’s activity and oxidant injury product of sows

Item	Dietary treatment		*P-*value
	Control	COS	
GSH-Px (U/mL)	629 ± 23.0	675 ± 11.2	0.105
T-SOD (U/mL)	72.7 ± 2.55b	81.6 ± 2.17a	0.027
CAT (U/mL)	4.19 ± 0.67	4.05 ± 0.64	0.857
MDA (nmol/mL)	3.95 ± 0.42	2.81 ± 0.38	0.069

Dietary treatment: control = basal diet, COS = basal diet + COS (30 mg/kg basal diet). Data are expressed as mean ± SEM, *n* = 8. ^a, b^Means values were significantly different compared with normal sows (*P* < 0.05). The same as below

### Antioxidant related gene expression and contents of total-SOD and catalase in placenta

**Fig. 1:**
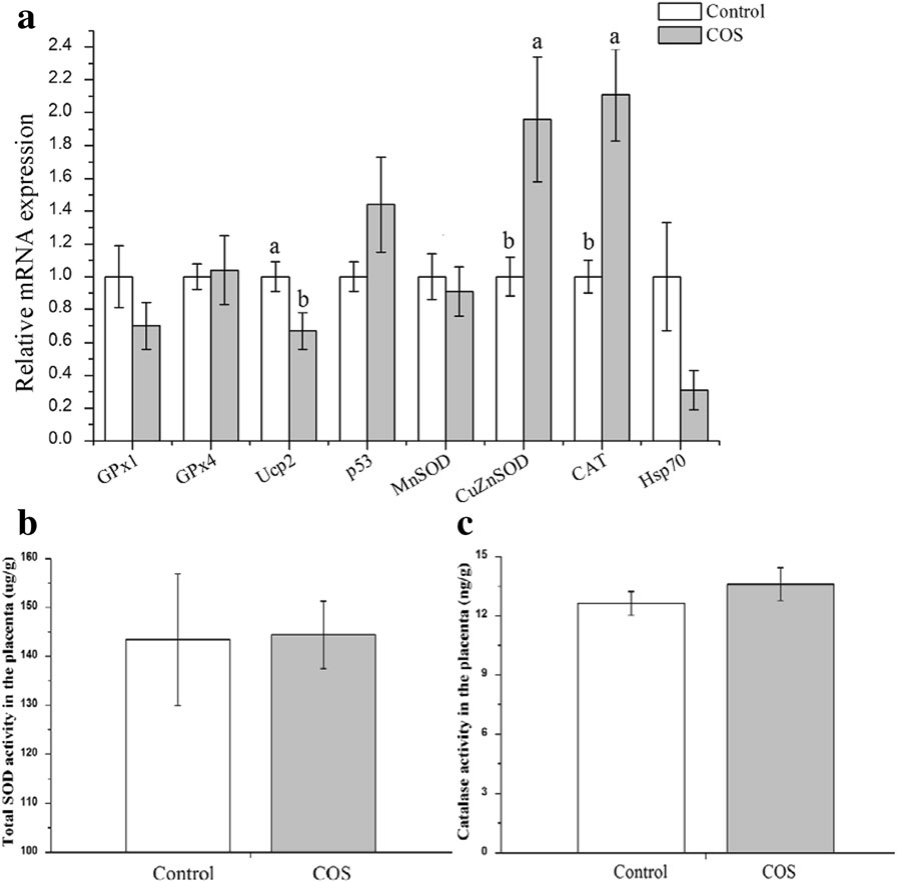
Oxidative stress-related genes expression (**a**), total SOD (**b**) and catalase (**c**) in the placenta of sows consuming control, COS diets during late gestation and lactation. Values are means (*n* = 8) with their standard errors represented by vertical bars

**Fig. 2:**
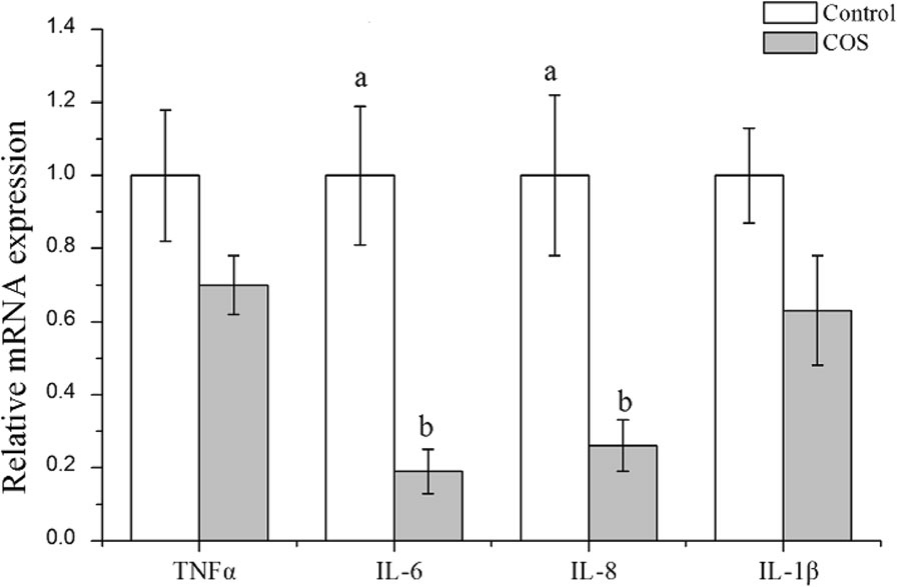
Pro-inflammatory cytokines expression in the placenta of sows consuming control, COS diets during late gestation and lactation. Values are means (*n* = 8) with their standard errors represented by vertical bars

To investigate the effect of dietary supplementation with COS on the oxidant stress and antioxidant capacity of placenta, we studied the mRNA expression of several anti-oxidant (Fig. 1(a)) or pro-inflammation cytokine (Fig. 2) genes in the placenta. The mRNA levels of anti-oxidant gene Cu/Zn-SOD (*P* < 0.05) and CAT (*P* < 0.01) were increased in the COS group, but Ucp2 was down-regulated (*P* < 0.05) compared with that in the control group. Meanwhile, COS supplementation resulted in decreased IL-6 (*P* = 0.001) and IL-8 (*P* < 0.05), or a downtrend (0.05 < *P* < 0.10) in Hsp70 and IL-1β, but no significant difference was observed (*P* > 0.10) in the mRNA levels of GPx1, GPx4, p53, TNFα and Mn-SOD between the two groups, which was consistent with the contents of total-SOD and CAT in the placenta (*P* > 0.10) (Fig. 1(b), (c)).

### Relative expression of amino acid transporters in placenta

**Fig. 3:**
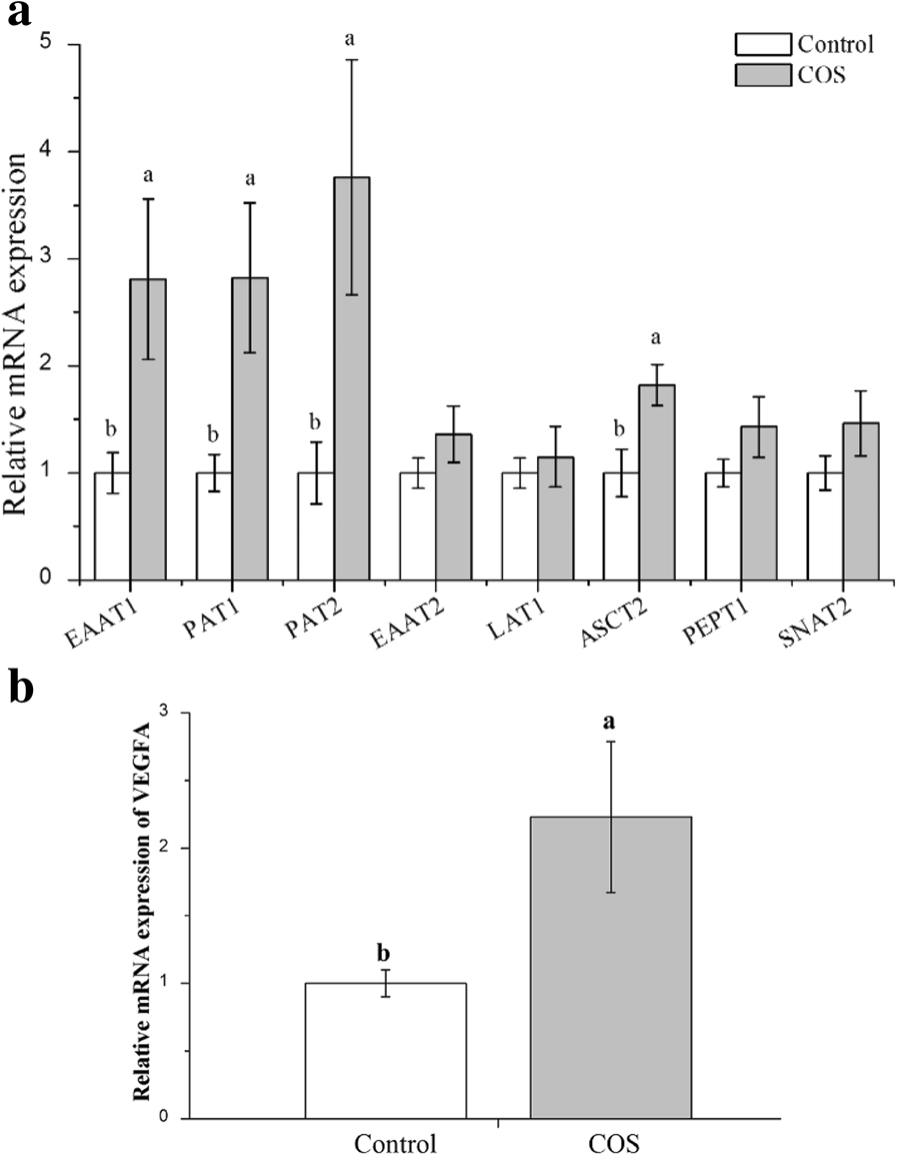
Amino acid transport-related genes (**a**) and VEGF-A expression (**b**) in the placenta of sows consuming control, COS diets during late gestation and lactation. Values are means (*n* = 8) with their standard errors represented by vertical bars

Except for the effect of COS on antioxidant defense in placenta, we also studied the relative expression of several amino acid transport-related genes (Fig. 3(a)). Compared with that in the control group, the mRNA levels of EAAT1, PAT1, PAT2 and ASCT2 in the COS group were 2.81, 2.82, 3.76 and 1.82-fold (*P* < 0.05), respectively. Noticeably, the mRNA level of vascular endothelial growth factor A (VEGF-A) in the COS group was 2.23-fold (*P* < 0.05) compared that in the control group (Fig. 2(b)). However, no significant difference was observed in mRNA expression of LAT1, EAAT2, PEPT1 and SNAT2 between the control and COS groups (*P* > 0.10).

### Relative protein expression of mTOR signaling pathway in the placenta

**Fig. 4:**
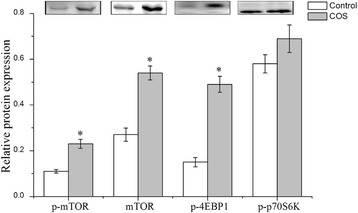
Relative protein expression of mTOR pathway in the placenta of sows consuming control, COS diets during late gestation and lactation. Values are means (*n* = 4) with their standard errors represented by vertical bars

It has been demonstrated intracellular or extracellular cues, such as amino acids, oxygen, and stress, could function to regulate mTOR pathway. In this study, the activity of the mTOR pathway was detected by measuring the protein abundances of p-mTOR, p-4EBP1, p-p70S6K and total mTOR. As shown in Fig. 4, mTOR signaling pathway in the placenta was stimulated. Compared with the control group, relative abundances of p-mTOR and total mTOR in placenta were increased (*P* < 0.05). In addition, the level of p-4EBP1, major downstream target of mTOR, was also up-regulated (*P* < 0.05), whereas the p-p70S6K level only exhibited an uptrend in the COS group (0.05 < *P* < 0.10).

## Discussion

It has been shown that pregnant sows had elevated oxidative stress during late gestation [18]. Oxidative damage is a strong indicator of health status and wellbeing of animals in relation to stress, nutritional status, and disease. Increased oxidative stress is responsible for impaired milk production, reproductive performance, and finally longevity of sows [4]. Antioxidant defenses can be enzymatic and non-enzymatic, and enzymatic defense includes SOD, CAT and GSH-Px, which act as a major part of antioxidant system [19].

Increased defense against oxidative stress of sows during gestation by supplementing with COS in diet was confirmed by an increase in total SOD activity. SOD is responsible for degrading superoxide radical into oxygen and hydrogen peroxide, which is then degraded by CAT. COS treatment could effectively increase the SOD level, a natural power that can be quickly used by the cells as a defense against oxidative stress [20]. Oxidative stress is described as an imbalance in the production of reactive oxygen species (ROS) and the ability of antioxidant defenses to scavenge them [1]. As the key ROS metabolic biomarker, MDA is from a series of reactions during lipid peroxidation caused by ROS [19]. The plasma MDA in the COS group, at least, partially was for the release of antioxidant enzymes, which scavenged excessive ROS. From the results of plasma analysis on d 110 of gestation, we concluded that COS supplementation could improve anti-oxidative capacity of pregnant sows.

The placenta appears particularly vulnerable to oxidative stress because of its extensive cell division and high metabolic activity [21]. ROS accumulation in placenta is limited by a range of major antioxidant, including Mn and Cu/Zn-SOD, CAT, GSH, GSH-Px, and vitamins C and E are present in the placenta [1]. Although these antioxidants, such as vitamins E, C and β-carotenes, could transfer via placenta during the last days of gestation, the antioxidant system is still insufficient to scavenge the excessive ROS [22]. The relative mRNA levels of Cu/Zn-SOD and CAT in the placenta were significantly increased by COS supplement, which was similar to the results of plasma MDA content and total SOD activity during gestation. However, inconsistent results were observed in the contents of total SOD and CAT, which may be explained that COS supplementation just increased the transcription level of some antioxidant genes in the placenta of sows without severe oxidative stress.

Previous study reported Ucp2-null mice showed increased ROS production in several cell types including macrophages [23] and adipocytes [24], indicating that mitochondrial Ucp2 may limit oxidative stress by reducing ROS production. It is unknown, however, whether Ucp2 plays a similar role in placental tissue remains to be established. Jones et al. (2010) found that dexamethasone reduced Ucp2 expression of placenta in the rat during late gestation in labyrinth zone [25], and is consistent with similar effects observed in bovine mammary epithelial cell cultures [26]. Consequently, the lower mRNA level of Ucp2 in the COS group may serve to enhance antioxidant protection in the late gestation placenta, and impact on placental nutrient metabolism, especially at times of increased energy demand [27].

Other than reduced Ucp2 expression, the major pro-inflammatory IL-6, IL-8, and IL-1β were decreased or presented a downtrend. Similar results were observed in rats [28], and mice [29]. Several studies reported that oxidative stress status in obese women during pregnancy was always associated with a pro-inflammatory state [2]. Hsp70 have been implicated in stress regulation, including heat shock, oxidative stress, and other environmental stresses [30]. In line with the relative expression of IL-6 and IL-8, the relative mRNA expression of Hsp70 in placenta had a downtrend in the COS group, which was consistent with previous study that activation of Hsp70 could mitigates inflammatory response of kidney [31]. These observation clearly indicated that COS supplementation in sow’s diet contributed reducing placental oxidative stress and inflammatory response, enhanced anti-oxidant defense capacity of placenta.

It has been demonstrated that mTOR is a central integrator of various signals, such as growth factors, nutrients, energy, and stress [32]. As an essential serine/threonine kinase, mTOR is a central regulator of cell growth and proliferation, which can directly phosphorylates the ribosomal p70S6K and the initiation factor 4E-BP1 to initiate translation of distinct mRNAs [33]. For example, stimulating mTOR signaling pathway could increase protein synthesis, and decrease protein degradation in porcine conceptus trophectoderm cells [34] and porcine enterocytes [35]. It has been shown that mTOR/4EBP1 could offer cellular protection during oxidant stress, and knockdown of 4EBP1 induces oxidative stress-induced apoptosis through a caspase-dependent pathway in human endothelial progenitor cells [36]. Consequently, the abundance of mTOR/4EBP1 may be the result of lower oxidative stress by COS supplementation in sow’s diet, and p70S6K being relative independent on the mTOR pathway in this study.

The mTOR pathway is regulated by a multitude of intracellular and extracellular signals, and is inhibited by hypoxia [37], then significantly reduces the activity of placental amino acid transporters [38]. SNAT2 and PAT are important amino acid transporters in stimulating mTOR pathway [39]. In the present study, we found that the relative mRNA levels of most of amino acid transporter EAAT1, PAT1, PAT2 and ASCT2 in the COS group were increased or presented uptrend compared with that in the control group, which was consistent with previous report that mTOR increased total amino acid transporter gene expression with enhanced surface expression in mouse lymphoma cells, and the great expression of amino acid transporter increased the mTOR activity [40]. These observations are in line with our hypothesis that the mTOR pathway in the placenta functions as a nutrient sensing pathway.

It has been shown that inhibiting the phosphorylation of mTOR down-regulated the PlGF mRNA level (PlGF, a member of the VEGF family proteins) [41]. In agreement with previous study, the relative mRNA level of VEGF-A in placenta was increased by COS supplementing. During pregnancy, VEGF-A protein increases in the maternal circulation during pregnancy in humans [42] and mice [43]. It has been shown that it was sufficient to cause embryonic lethality when the mRNA level of VEGF-A fall by half [44]. Furthermore, VEGF-A gene deletion in the conceptus itself may result in alteration placental gene expression, reduced fetal body weight, and higher maternal cardiac output [45]. In this study, COS supplement significantly increased the VEGF-A mRNA level in placenta, indicating that supplementation with COS in sow diets could affect fetal development by stimulating mTOR pathway, involved in VEGF-A.

## Conclusion

In summary, these observations suggested that maternal dietary supplementation with COS protected sows against oxidative stress by increasing plasma antioxidants and blocking inflammatory response, which may contribute to promoting placental amino acids transport by activating mTOR signaling pathway. Consequently, COS supplementation during late gestation may be an effective means in conforming the health of pregnant sows and nutrition transport from sows to fetus.

## Abbreviation

4E-BP1: The initiation factor 4E-binding protein 1
ASCT2: Alanine-serine-cysteine-threonine transporter
CAT: Catalase
COS: Chitosan oligosaccharide
EAAT: Glutamate transporter
GSH-Px: Glutathione peroxidase
LAT1: L-type amino-acid transporter 1
MDA: Malondialdehyde
mTOR: Mammalian target of rapamycin
p70S6K: Phosphorylates the ribosomal p70S6 kinase
PAT: Proton-assisted amino acid transporter
PEPT: Peptide transporter
Per1: Negative-regulated element period 1, CLOCK, circadian locomotor output cycles kaput
ROS: Reactive oxygen species
SNAT: Sodium-coupled neutral amino acid transporter
T-SOD: Total superoxide dismutase
Ucp2: Uncoupling protein
VEGF-A: Vascular endothelial growth factor A
